# Scattered Radiation Emission Imaging: Principles and Applications

**DOI:** 10.1155/2011/913893

**Published:** 2011-06-07

**Authors:** M. K. Nguyen, T. T. Truong, M. Morvidone, H. Zaidi

**Affiliations:** ^1^Laboratoire Equipes Traitement de l'Information et Systèmes, CNRS UMR 8051/ENSEA, Université de Cergy-Pontoise, 95302 Cergy-Pontoise, France; ^2^Laboratoire de Physique Théorique et Modélisation, CNRS UMR 8089, Université de Cergy-Pontoise, 95302 Cergy-Pontoise, France; ^3^Facultad Regional Buenos Aires, Universidad Tecnológica Nacional, Mozart 2300, C1407IVT Buenos Aires, Argentina; ^4^Division of Nuclear Medicine, Geneva University Hospital, 1211 Geneva 4, Switzerland; ^5^Geneva Neuroscience Center, Geneva University, 1211 Geneva 4, Switzerland

## Abstract

Imaging processes built on the Compton scattering effect have been under continuing investigation since it was first suggested in the 50s. However, despite many innovative contributions, there are still formidable theoretical and technical challenges to overcome. In this paper, we review the state-of-the-art principles of the so-called scattered radiation emission imaging. Basically, it consists of using the cleverly collected scattered radiation from a radiating object to reconstruct its inner structure. Image formation is based on the mathematical concept of compounded conical projection. It entails a Radon transform defined on circular cone surfaces in order to express the scattered radiation flux density on a detecting pixel. We discuss in particular invertible cases of such conical Radon transforms which form a mathematical basis for image reconstruction methods. Numerical simulations performed in two and three space dimensions speak in favor of the viability of this imaging principle and its potential applications in various fields.

## 1. Introduction

Since the early 50s, ionizing radiations (in particular gamma-rays), because of their penetrating property, have been used to explore the interior of objects. At first, this was done in transmission mode with an external radiation source, which projects a shadow onto a plane detector. Later, it was shown that a three-dimensional image can be reconstructed provided there is a sufficient number of such two-dimensional projections generated by the displacement of the source/detector pair in space. This three-dimensional image reconstruction of the inner object structure relies on the existence of the inverse of the so-called X-ray transform, which correctly models the above process of data acquisition [1, 2].

A second modality, called emission imaging, deals with radiation-emitting objects. In nuclear medicine, this modality concerns objects (human organs), which, after injection of a radiotracer, displays its biodistribution in the human body. Nowadays, the image reconstruction of both single-photon emitting and positron-emitting tracer distributions is achieved by single-photon emission computed tomography (SPECT) [3] and positron emission tomography (PET) [4, 5], respectively. These two modalities are based on the invertibility of the standard Radon transform.

However, due to the interaction of radiation with matter, gamma-ray imaging is plagued by Compton scatter, which degrades image quality and spatial resolution. Thus, effects due to scattered photons should at best be eliminated or at least be reduced [6]. However, a more astute point of view would be to take advantage of their properties either for improving image quality or for generating new imaging processes. The idea of Compton scatter imaging has been launched many years ago and many ways to exploit Compton scattering for imaging purposes have been introduced.

An early proposal goes back to the 50s [7], but interest in this concept has remained vivid [8], because this idea has many highly desirable features. In the field of diagnostic medical imaging, radiography using scattered radiation could provide a direct and quantitative measurement of the density of the studied object. In nondestructive testing, it offers three advantages.

- It permits to place both the radiation source and the detector on the same side of the object.
- It has also greater sensitivity to low-density materials such as gases.
- Finally, it allows direct spatial definition with high-contrast resolution.

With the advent of X-ray computed tomography (CT), interest in Compton scatter imaging has waned for a while. But research in this field has remained very much alive, and a large variety of imaging techniques have been developed [3, 9, 10]. 

Earlier modalities for Compton scatter imaging are classified according to the way measurement of the spatial distribution of scattered radiation is done or the number of simultaneous volume elements being scanned: that is, point by point, line by line, or plane by plane (see reviews [9, 10]). Most of the devices work at constant scattering angles (generally at 90 degrees). In the mid-90s, the concept of Compton scatter tomography was introduced by Norton [11] and subsequently developed by many other workers [12, 13]. A prominent example in which Compton scattering acts as imaging agent without mechanical collimation is the co-called Compton camera [14–16], as well as gamma-ray tracking imaging or the like. More recently, Compton scatter imaging using annihilation pair photons with coincidence measurements has appeared on the scene as a yet unexploited imaging technique [17]. Related concepts allow enhancing the detection efficiency by reconstructing a significant fraction of events which underwent Compton scattering in crystals [18].

In this work, we review a different approach to Compton-scattered radiation for * emission* imaging. We first point out why scattered photons should be used instead of primary photons in gamma-ray imaging. Then, we show how the image formation process is actually performed using the concept of compounded conical projection. The basic mathematical object to be considered is a generalized Radon transform on circular cone surfaces.  Section 2 describes the general principles of this imaging modality and its properties. Then, in consecutive subsequent sections, we treat explicitly a special case, involving a standard gamma camera-based SPECT system, in two and three dimensions. Results on numerical simulations are also presented; they speak in favor for the realizability of this imaging method, once real world issues (noise, energy uncertainty, sampling, etc.) are addressed and resolved. A conclusion summarizes this paper and points out future research perspectives. 

## 2. The Emission Imaging Problem

The aim is to image the interior of radiating (or made radiating) objects, which emit gamma-rays of a given energy *E*_0_ resulting from a nuclear transition, (e.g., Technetium 99 m in nuclear medicine with *E*_0_ = 140 keV). To intercept the emitted gamma photons, we will use a device called a *directional detector* (or DD for short). This is a point-like pixel at a site **D** in space, capable of absorbing gamma photons of any energy *E* below *E*_0_, in a given direction specified by a unit vector **n**. It may be thought as a one-pixel collimated gamma camera. This device is only used for argumentation.

**Figure 1:**
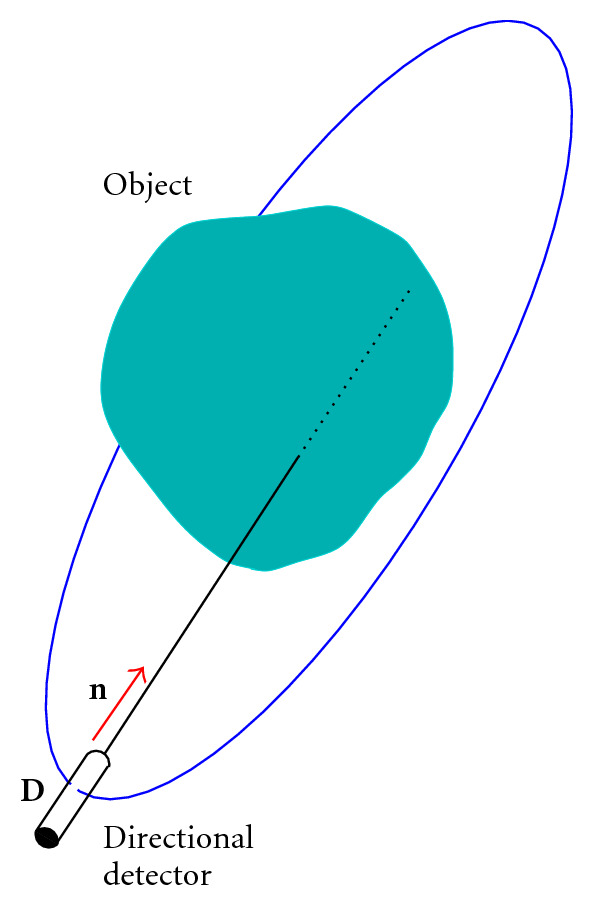
Linear projection through an object.

For each pair (**D**, **n**), the DD records a photon flux density coming directly from the radiating object reaching the pixel site **D** in the direction −**n**. This measurement is called in the specialized jargon a *projection*, or better a *linear projection*, since it is done along a straight line passing through **D** in the direction **n**. As the recorded photons carry the original energy *E*_0_, they are called primary (or nonscattered) photons. In this way, the set of such measurements constitutes the conventional X-ray projection data of the object radioactivity function, which is represented by a nonnegative, well-behaved, and compactly supported function *f*(**r**) = *f*(*x*, *y*, *z*). Mathematically, this data is represented by *Xf*(**D**, **n**), the so-called X-ray transform of *f*(*x*, *y*, *z*).  Figure 1 illustrates this measurement concept.

As the X-ray transform is invertible, albeit under conditions, *f*(**r**) can be obtained by various reconstruction algorithms (see, e.g., among the many references [21–24]). 

### 2.1. Scattering

**Figure 2:**
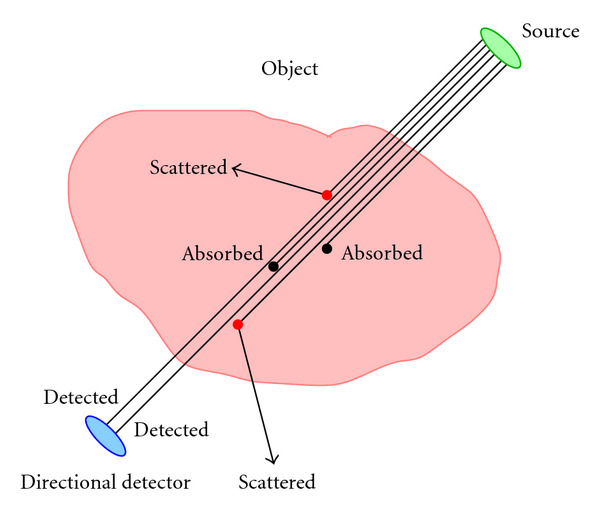
Illustration of the problem of photon attenuation resulting from photoelectric absorption and Compton scattering.

Photon transport through matter suffers from two lossy phenomena: photoelectric absorption and Compton scattering (Figure 2). The net depletion in photon number is described by a macroscopic coefficient of linear attenuation of the traveling photon flux density. However, in the energy range of a few hundred keV, Compton scattering is dominant and photoelectric absorption is negligible in biological tissues. Scattered photons have a disturbing effect in nuclear medicine imaging, see, for example, [25]. It causes blurring, loss of contrast, and false detection of emitting sources in the context of primary (nonscattered) radiation imaging. So it is natural to raise the question: is scattered radiation any good for imaging?

**Figure 3:**
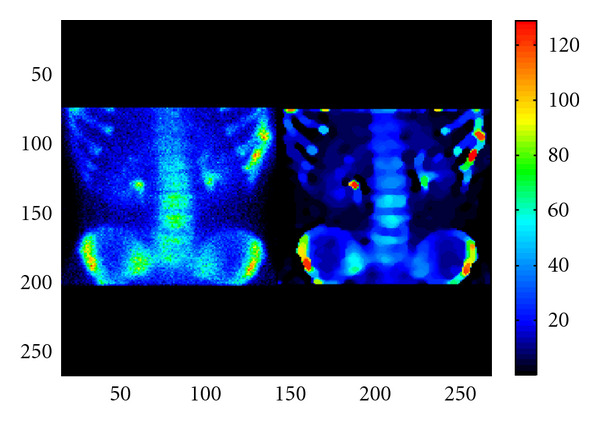
Bone scintigraphy: a standard scintigraphic image (left image) is restored with the use of Compton-scattered radiation. Hot spots or nodule are clearly displayed (right image—reprinted with permission from [19]).

Handling away scatter in gamma-ray imaging has been pioneered by many authors. Algorithms to compensate for Compton scattering in SPECT imaging have been developed, for example, [26], and techniques allowing the determination of source depth via scattered radiation proposed [27]. The idea of using scattered photons to reduce the noise level of SPECT images has emerged in [28], in which data acquired in the photopeak and various scatter energy windows are statistically assembled to improve image quality. In 2001, it was observed that scattered radiation images of an object may be sorted out at a given energy (or at a given wavelength) using standard gamma camera data operating in list mode [29]. A series of apparent images labeled by the photon-scattered energy of the object is then acquired [30]. Subsequently, they are taken into account in the process of image restoration. Small details, unresolved before, emerge clearly separated from each other. As an example in bone scintigraphy, Figure 3 shows that small hot spots or nodules, which are invisible on the left image, become perfectly distinguishable on the right image after accounting for scattered radiation. This is, in fact, very valuable for clinical diagnosis, assessment of response to treatment, and radiation therapy treatment planning. Thus, the newly revealed resolving power brought by scattered radiation has appeared very attractive for further development. 

Therefore, it is of interest to take a close look at the effect of scattering in photon detection. Let us consider first the case of a point source emitting a monochromatic red light by a clear day. A human eye, placed at a certain distance from this source, would see only a red spot. However, if a fog cloud sets in, then the eye would see a diffuse red cloud much larger than the red spot. The fog cloud has made itself visible because the emitted red light is scattered by the fog droplets and re-emitted as scattered light by the fog droplets acting as scattering centers. This fog cloud has become a kind of secondary radiating object, visible to the human eye. It also implies the existence of a concealed red light source.

In the gamma energy range, a similar observation can be made. A single gamma-ray emitting point source behaves exactly in the same way with respect to our directional detector (DD), which is a kind of gamma-ray sensitive “eye.” But if the gamma-ray emitting point source is embedded in a medium of finite volume, which plays the role of the fog cloud, then light wave scattering by water droplets is replaced by Compton scattering of emitted photons with electric charges of the surrounding medium. If visible light emerges from scattering without changing its wavelength (*Rayleigh scattering*), the emerging scattered gamma ray has an energy *E* lower than the incident energy *E*_0_, because part of *E*_0_ is transferred to electric charges in the traversed medium. As *E* is continuously distributed, the gamma-ray sensitive eye (DD) would now “see” a *red-shifted “polychromatic” radiation* emanating from the scattering medium volume [20]. The wavelengths *λ* of the scattered gamma-rays are longer than the incident wavelength *λ*_0_, as given by the Compton formula [31],

(1) $$\lambda =\lambda_{0}\;+\frac{h}{m_{e}c}\;\left(1-\cos\;\;\omega \right),$$

where *m*_*e*_ is the electron mass, *h* is the Planck constant, *c* is the speed of light in vacuum, and *ω* is the Compton scattering angle.

Now instead of having a single-point source, consider a nonuniform three-dimensional distribution of gamma-ray emitting point sources embedded in a medium of finite volume. The question is what flux density of photons of energy *E* < *E*_0_ would a DD record at a site **D** in a direction **n**? To give an answer, we should follow the backward track taken by the gamma-ray before it reaches the DD.

**Figure 4:**
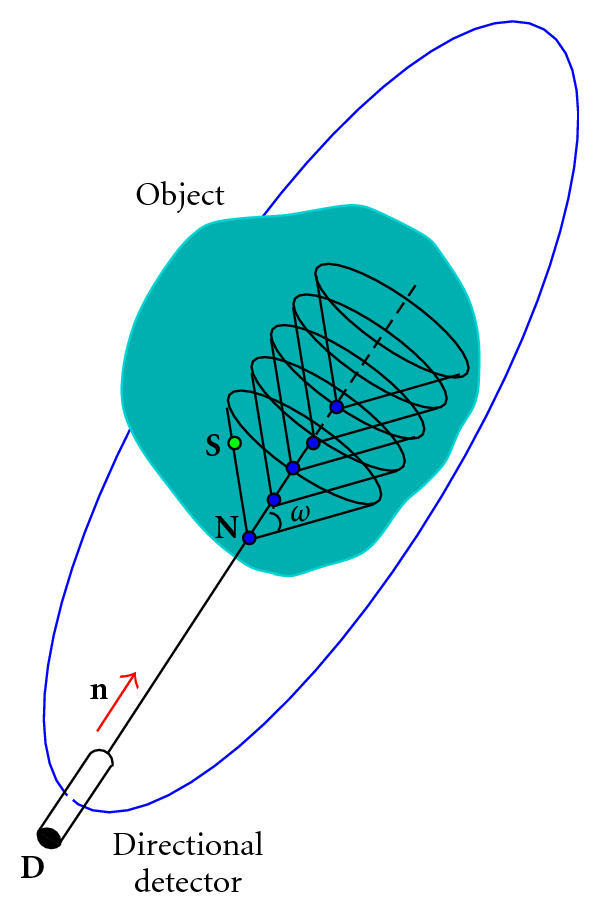
Compounded conical projection.

A gamma photon arriving at **D** with energy *E* must have gone through a scattering with some electric charge at a site **N** situated on the straight line, which starts from **D** in the direction of **n**, see Figure 4. As the scattering angle *ω* is given by the Compton formula

(2) $$E=E_{0}\;\frac{1}{1-\varepsilon \;\cos\;\omega },$$

where *ε* = *E*_0_/*m*_*e*_  *c*^2^, this photon must have originated from a site **S**, located on the surface of a circular cone of vertex **N**, axis *DN*, and opening angle *ω*. Following this picture, we can write down the photon flux density detected at **D**. 

To this end, we need the expression of the Compton differential cross-section, which reads as the product of the electron density *n*_*e*_(**N**) at the scattering site **N** by the so-called Klein-Nishina probability *𝒫*(*ω*) [25, 31], that is, *n*_*e*_(**N**)*𝒫*(*ω*),

(3) $$\begin{array}{cc} 𝒫\left(\omega \right) & =\pi r_{e}^{2}\;\frac{1}{2\pi }\frac{1}{{\left[1+\varepsilon \left(1-\cos\;\;\omega \right)\right]}^{2}}\\ & \;\times \left(1+{\cos\;}^{2}\omega +\frac{\left(1-{\cos\;}^{2}\omega \right)}{1+\varepsilon \left(1-\cos\;\;\omega \right)}\right). \end{array}$$

Radiation emitted at point source **S** is assumed to be isotropic. It propagates then to scattering site **N** and reaches the detection site **D** at the end. The incoming photon flux density on scattering site **N** is computed from the emission data at a point source. Let *f*(**S**)  *d ***S** be the number of gamma photons emitted per unit of time by a volume *d ***S** in the object around site **S**. The emission being isotropic, the number of photons emitted in the direction $\vec{SN}$ in a solid angle *dΩ*_*S*_ is

(4) $$\frac{f\left(S\right)dS}{4\pi }\;d\Omega_{S}.$$

Therefore, the incoming photon flux density at scattering site **N** is given by

(5) $$\frac{f\left(S\right)dS}{4\pi }\;\frac{e^{-𝒜(SN)}}{SN^{2}},$$

where $SN=|\vec{SN}|$ and *e*^−*𝒜*(*SN*)^ is the attenuation factor along the path *SN* and is given by the integral

(6) $$𝒜\left(SN\right)=\overset{N}{\underset{S}{\int }}ds\mu \left(S+sk\right),$$

where $k=\vec{SN}/SN$ and *μ*(**M**) is the linear coefficient of absorption. Recall that, by assumption, *μ* is equal to the product of the Compton scattering cross-section and the electron density. 

Next, the number of scatterers around site **N** in a volume *d ***N** is *n*_*e*_(**N**)  *d ***N**. The net number of photons emerging from the scattering in an elementary solid angle *dΩ*_*N*_ is

(7) $$\frac{f\left(S\right)dS}{4\pi }\frac{1}{SN^{2}}n_{e}\left(N\right)dN\pi r_{e}^{2}𝒫\left(\omega \right)d\Omega_{N}.$$

This means in turn that the detected photon flux density at site **D** is

(8) $$\frac{f\left(S\right)dS}{4\pi }\;\frac{e^{-𝒜(SN)}}{SN^{2}}n_{e}\left(N\right)dN\pi r_{e}^{2}𝒫\left(\omega \right)\frac{e^{-𝒜(ND)}}{ND^{2}}.$$

Now, all the contributing point sources **S**, for a given scattering center **N**, lie on a circular cone sheet of axis identified with $\vec{DN}=n\;DN$ and opening angle *ω*. Thus, we must integrate with the measure *δ*(cone )*d ***S** first. Next, we must take into account all the scattering sites on the line *DN*. Hence, we must perform a second integration with the measure *δ*(line)*d ***N**. Consequently, the detected photon flux density at **D** in the direction **n** for a scattering angle *ω* is

(9) $$\begin{array}{cc} \hat{f}\left(D,n,\omega \right) & =\int \int \delta \left(\mathrm{cone}\;\right)\frac{f\left(S\right)dS}{4\pi }\;\frac{e^{-𝒜(SN)}}{SN^{2}}\\ & \;\times n_{e}\left(N\right)dN\pi r_{e}^{2}P\left(\omega \right)\delta \left(\text{line}\right)\frac{e^{-𝒜(ND)}}{ND^{2}}, \end{array}$$

where *δ*(cone ) means the delta function concentrated on the cone of vertex **N**, opening angle *ω*, and axis $n=\vec{DN}/DN$; *δ*(line) is the delta function concentrated on the line *DN*.

$\hat{f}(D,n,\omega )$ will be called compounded conical projection data in the direction **n**, at site **D**, and with given scattering angle *ω* [20]. $\hat{f}(D,n,\omega )$ is also called the compounded conical Radon transform (CCRT) of *f*(**r**), a generalized Radon transform on circular cone surfaces as originally introduced in [32].

 Assuming that exchange of integration is valid, we may view $\hat{f}(D,n,\omega )$ as

(10) $$\begin{array}{cc} \hat{f}\left(D,n,\omega \right) & =\pi r_{e}^{2}𝒫\left(\omega \right)\int dN\delta \left(\text{line}\right)n_{e}\left(N\right)\frac{e^{-𝒜(ND)}}{ND^{2}}\\ & \;\times \int \frac{f\left(S\right)dS}{4\pi }\delta \left(\mathrm{cone}\;\right)\frac{e^{-𝒜(SN)}}{SN^{2}}. \end{array}$$

This result may be regarded as the X-ray transform (on the straight line passing through **D** in the direction **n**) of the function

(11) $$g\left(N\;|\;n,\omega \right)=n_{e}\left(N\right)\frac{e^{-𝒜(ND)}}{ND^{2}}\int \frac{f\left(S\right)dS}{4\pi }\delta \left(\mathrm{cone}\;\right)\frac{e^{-𝒜(SN)}}{SN^{2}},$$

or

(12) $$\hat{f}\left(D,n,\omega \right)=\pi r_{e}^{2}𝒫\left(\omega \right)Xg\left(N\;|\;n,\omega \right).$$

So by letting the directional detector (DD) take all possible spatial orientations, we generate the totality of possible compounded conical projections, which depends on five parameters: four for a line in ℝ^3^ and one for the scattering angle (*ω*). In principle, the object under study is the support for two functions *f*(**r**), the object radioactivity distribution and *n*_*e*_(**r**) its electron density distribution to be simultaneously determined. This problem is akin to the identification problem in the exponential Radon transform [33]. It seems to be difficult to solve at present since, by inspection, the DD data is underdetermined although it would be in principle possible to perform the inversion of the X-ray transform in (12), to retrieve *g*(**N** | **n**, *ω*). Thus, in the coming sections, we will review two major achievements of the compounded conical Radon transforms in ℝ^*n*^ with *n* = 2,3 and discuss the properties of the corresponding imaging modalities. 

## 3. Compton-Scattered Radiation Imaging in Three Dimensions

### 3.1. The Compounded Conical Radon Transform (CCRT)

In this section, we examine a tractable case by which a particular set of compounded conical projections is used for image reconstruction. This is possible under the following conditions:

- First-order scattering events are accounted for since they are vastly dominant and higher-order scattering are neglected [6, 34],
- the electron density is assumed to be constant. This is a reasonable hypothesis since most human tissues (brain cells, blood, muscles, lung tissues, water, etc.) have an electron density around 3.4 × 10^23^ cm^−3^. Their density is also around 1.0 g·cm^−3^ [19]. This means for our purpose, objects containing bones should not be considered,
- the set of compounded conical projections has one fixed direction **n**, parallel to the *Oz* axis direction,
- the set of detecting pixels are distributed as array on a two-dimensional area, forming a collimated SPECT gamma camera.

**Figure 5:**
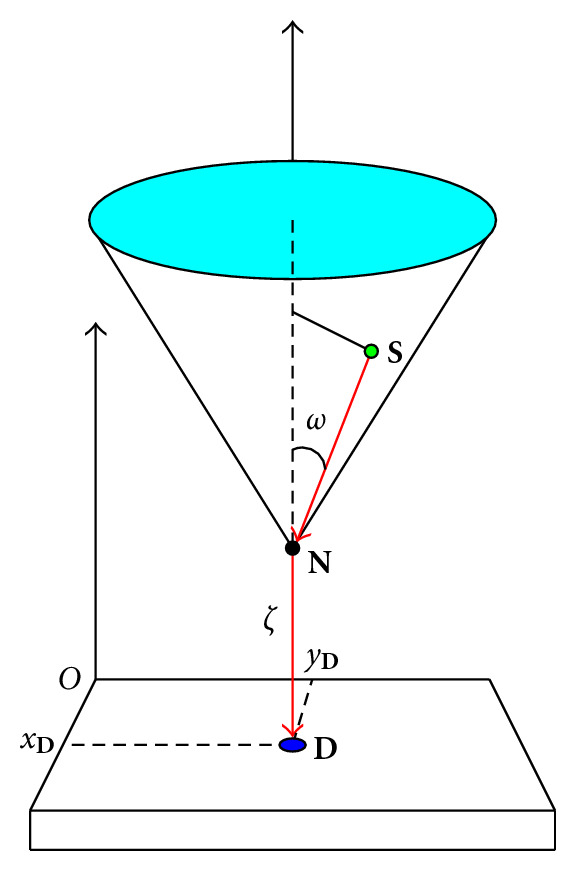
Coordinate system used in CCRT computation.

Using the coordinate system of Figure 5 and following the path of a photon from emission to absorption via one scattering at a site **N**, the expression of the flux density on the detector at a site **D** = (*x*_*D*_, *y*_*D*_, 0), $\hat{f}(x_{D},y_{D},\omega )$ has the form of a linear integral transform of the object activity density *f*(*x*, *y*, *z*),

(13) $$\begin{array}{cc} & \hat{f}\left(x_{D},y_{D},\omega \right)\\ & \;\;=\int_{{\mathbb{R}}^{3}}dx\;dy\;dz{𝒦}_{\text{PSF}}\left(x_{D},y_{D},\omega \;|\;x,y,z\right)\bar{f}\left(x,y,z\right), \end{array}$$

where the function $\bar{f}(x,y,z)$ is defined by

(14) $$\bar{f}\left(x,y,z\right)=\overset{\infty }{\underset{0}{\int }}d\zeta \nu \left(\zeta \right)f\left(x,y,z+\zeta \right),$$

and the integration kernel is

(15) $$\begin{array}{cc} & {𝒦}_{\text{PSF}}\left(x_{D},y_{D},\omega \;|\;x,y,z\right)\\ & \;=K\left(\omega \right)\nu \left(\sqrt{{\left(x-x_{D}\right)}^{2}+{\left(y-y_{D}\right)}^{2}+{\left(z-\zeta \right)}^{2}}\right)\\ & \;\;\times \delta \left(\cos\;\;\omega \sqrt{{\left(x-x_{D}\right)}^{2}+{\left(y-y_{D}\right)}^{2}}-\left(z-\zeta \right)\sin\;\omega \right), \end{array}$$

*K*(*ω*) is the Compton kinematic factor, and *ν*(*d*) is a function describing a photometric factor for a distance *d*, for example, *ν*(*d*) = 1/*d*^2^. By definition, $\hat{f}(x_{D},y_{D},\omega )$ is called the compounded conical Radon transform (CCRT) of *f*(*x*, *y*, *z*).

The adopted working hypotheses are aimed to avoid unnecessary complications which would mask the main idea.

The inversion of the kernel *𝒦*_PSF_(*x*_*D*_, *y*_*D*_, *ω* | *x*, *y*, *z*) is then obtained via a form of central slice theorem in Fourier space of the detector plane for the function $\bar{f}$, followed by a deconvolution to get the Fourier transform $\tilde{f}(u,v,w)$ of *f*(*x*, *y*, *z*), see [32, 35],

(16) $$\begin{array}{cc} & \tilde{f}\left(u,v,w\right)\\ & =\int_{\mathbb{R}}d\sigma \exp\;\left[2i\pi \sigma w\right]\frac{\left[-\left|z\right|\sqrt{u^{2}+v^{2}}\right]}{J\left(w\right)}\;\\ & \;\times \int_{{\mathbb{R}}_{+}}t\;dtJ_{1}\left(2\pi \left|z\right|t\sqrt{u^{2}+v^{2}}\right)\\ & \;\times \left[H\left(\omega -\frac{\pi }{2}\right)\frac{\partial }{\partial t}\frac{G\left(u,v,t\right)}{K\left(t\right)}+H\left(\frac{\pi }{2}-\omega \right)\frac{\partial }{\partial t}\frac{G\left(u,v,-t\right)}{K\left(-t\right)}\right], \end{array}$$

where *J*(*w*) is the Fourier transform of *ν*(*x*), *J*_1_(*x*) is the Bessel function of order 1, *H*(*x*) is the Heaviside unit step function, *t* = tan*ω*, *G*(*u*, *v*, *t*) is the Fourier transform of *g*(*x*_*D*_, *y*_*D*_, *t*), *K*(*t*) is the Compton kinematic factor as a function of *t*.

Finally, *f*(*x*, *y*, *z*) is recovered by three-dimensional inverse Fourier transform. It is observed that the data acquisition can be performed with a *spatially fixed* SPECT camera operating at successive scattered energies. As the scattering angle parameterizes series of “images” of the object, one may view it as replacing the spatial rotation angle in a standard SPECT data acquisition. This is a major advantage offered by this approach. 

For practical purposes, the effective treatment of (16) is in itself a numerical challenge. It has been fully done in chapter 4 and appendices *A* and *B* of [19], where details on samplings in object space and medium space are given. Because the kernel of (16) is a Bessel function of order zero, to control the oscillations, an exponential discretization step is to be used as suggested in [36]. Along the line perpendicular to the planar detector, the high-frequency components of the activity function information are carried by the weakly scattered (or back-scattered) radiation, the sampling step should then be very small. But for the low-frequency components of the activity function, it is the strongly scattered photons (*ω* ~ *π*/2) which carry information; the sampling step should be then much larger. For a primary radiation energy of 140 keV, to obtain the same spatial resolution, the detector should have a very fine energy resolution Δ*E*. In fact, simulations show that the activity function reconstruction is satisfactory for Δ*E* ~ 0.5 keV (even in the presence of a 24 dB white noise). The key point is that a reconstruction of reasonable quality can be achieved using the inverse CCRT. 

### 3.2. Point Spread Function of the CCRT and Simulation Results

**Figure 6:**
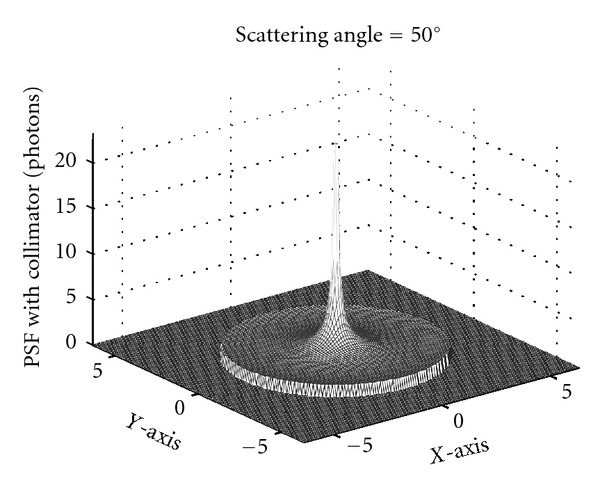
The PSF at a scattering angle of 50 degrees with collimator (reprinted with permission from [20]).

A way to get an idea of what the CCRT could be or do is to construct its point spread function (PSF), or the response function to a unit point source. It does not have the form of a delta-function as in the usual Radon transform. Because of the integration over all cones standing on top of the detecting site **D**, it appears as a function with the shape of a Mexican hat as shown in Figure 6; the point source is located somewhere on the vertical line symmetry axis of the Mexican hat above the planar detector [35].

The gamma detector operates now at a *fixed position*. No coincidence detection, as in Compton cameras, is required. Performed numerical simulations are in favor of the feasibility of this new imaging principle [35]. However issues related to higher-order scattering contribution, nonuniform attenuation, Poisson emission noise, detection sensitivity, and collimator efficiency are to be resolved before interesting practical modalities with possible combination with transmission imaging can be proposed [37].

To provide more convincing arguments regarding the viability of this idea, we present numerical simulations which illustrate the reconstruction of a simple cylindrical object using the analytic inversion formula with the following working conditions:

- the used *γ*-detector is a conventional SPECT camera. It has discretized dimensions *N* length units × *N* length units. The length unit is arbitrary but should remain coherent with reality and in fact is taken equal to 1 mm. We have chosen *N* = 16 to keep the calculations required at a reasonable level,
- the scattering medium is represented by a cube of dimensions *N* × *N* × *N* (length unit)^3^,
- the electron density in biological medium is *n*_*e*_ = 3.5 × 10^23^ electrons/cm^3^,
- the radionuclide employed is Tc-99 with an activity concentration corresponding to 4.84 × 10^10^ counts per minutes per cm^3^,
- the acquisition time per projection is set to 0.1 sec,
- the 3D original object (a cylinder of height 6 arbitrary units) is placed at the center of the scattering medium (cube),
- the distance from the camera to the upper face of the scattering medium cube is *l* = 200 arbitrary length units.

**Figure 7:**
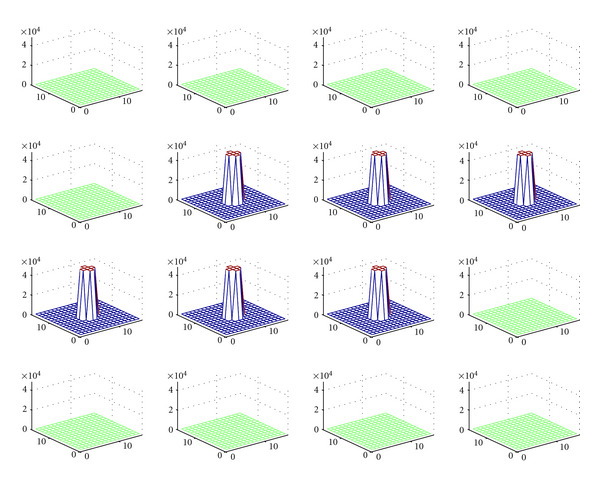
Original object (cylinder) in a cube consisting of 16 transaxial planes (reprinted with permission from [20]).

**Figure 8:**
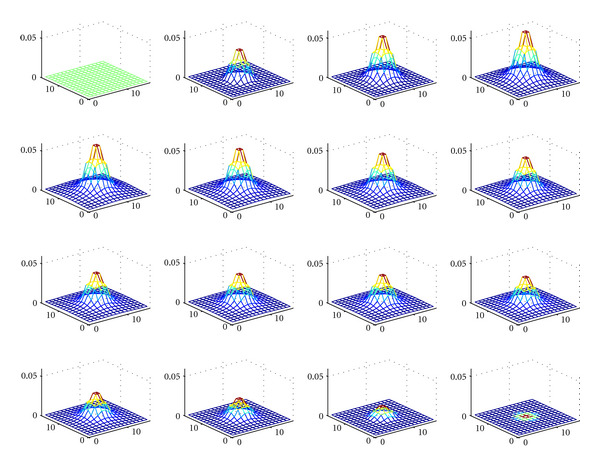
Series of images parameterized by the angle of Compton scattering *ω*(5° < *ω* < 175°) (reprinted with permission from [20]).

**Figure 9:**
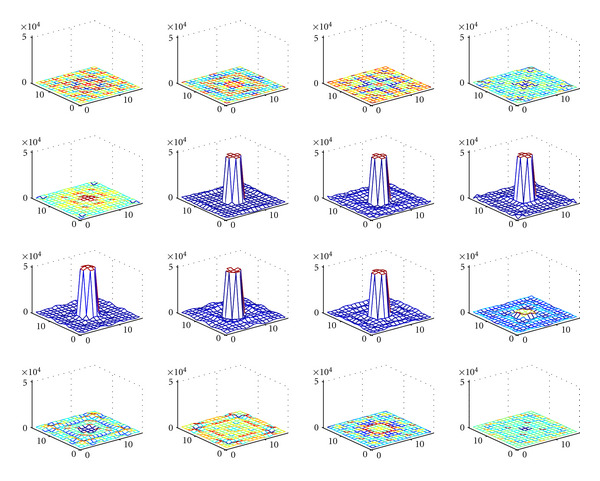
Reconstructed object in the absence of noise (RMSE = 1.2%) (reprinted with permission from [20]).

Figure 7 represents the original object.  Figure 8 shows the series of images of the object at various scattering angles *ω* (5° < *ω* < 175°). In Figure 9, the reconstructed object in the absence of noise is illustrated with a relative mean square error (RMSE) = 1.2%, which is perfectly reasonable. We observe a good performance of the CCRT for modeling the new imaging process.

Concerning spatial resolution, the intrinsic resolution depends on the camera design (collimator, crystal, photomultiplier tubes, and measurement electronics). The reconstructed system resolution is further determined by the reconstruction algorithm used. The inclusion of scattered radiation increases considerably the number of detected photons, which might contribute to improve the signal-to-noise ratio (SNR) and the resolution of the imaging system. To evaluate accurately the spatial resolution, it is necessary to use real data and to compare it with conventional methods which do not make use of scattered radiation. At the present time, it is too early to use our preliminary simulation results for this purpose.

Since our main objective in this paper is to show how to exploit advantageously Compton-scattered radiation to propose a new imaging principle, we focus on results illustrating the image formation process as well as image reconstruction from scattered photons.

In real situations, of course, one must take into account other factors such as photon attenuation by the medium, Poisson emission noise, and imperfections of the detector system including the collimator and electronics.

The case of uniform attenuation (often assumed in the literature) was included in [35]. The exact treatment of inhomogeneous attenuation poses enormous mathematical difficulties. Concerning emission noise, several approaches have been suggested to deal with it such as maximum likelihood or wavelets method. They may be used for “denoising” the measured data beforehand or jointly with the inversion process.

As for the imperfections of the detector, the standard way for treating this problem is to make use of a response function usually modeled as a Gaussian defined both in spatial and energy coordinates. These issues are discussed in details in [19, 29]. 

### 3.3. A Possible Generalization and Its Numerical Test

**Figure 10:**
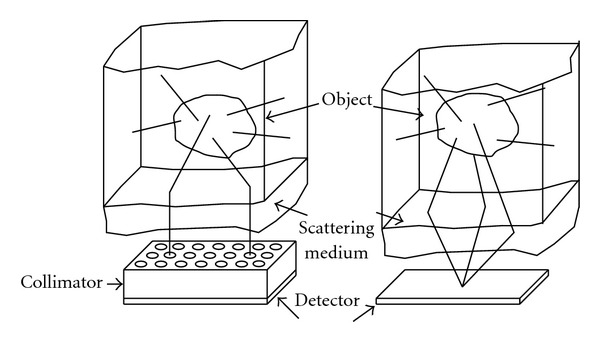
Two imaging modalities using Compton-scattered radiation with and without collimation (reprinted with permission from [20]).

As mentioned earlier, the presence of a mechanical collimator restricts severely the sensitivity of the imaging process. We have recently advocated a new functional modality following the principle of emission imaging by scattered gamma-rays without mechanical collimation [37, 38]. Removing the collimator from the detector allows more gamma-rays to reach a detecting pixel from all directions coming from the upper half-space of this site, therefore increasing the strength of the signal (Figure 10). An introductory study in two dimensions has recently been performed [39] to show convincingly the viability of this idea and to motivate the present work.

**Figure 11:**
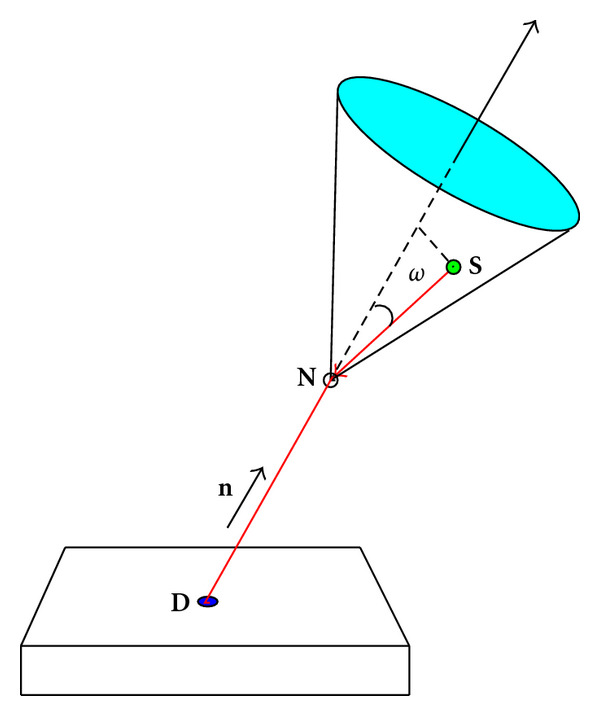
A conical projection in the gCCRT.

The modeling of the image formation process is done by a more general compounded conical Radon transform, whereby one sums over conical projections at one detection pixel over all cone vertices in the upper half-space.  Figure 11 shows the position of one conical projection in this generalized gCCRT. 

This transform is obtained by summing over all scattering sites for a given site **D** on the detector. The mathematical expression of one arbitrary conical projection is quite involved and given in [40]. The summation over all such objects can be still expressed as a linear integral transform of the activity density *f*(*x*, *y*, *z*),

(17) $$g\left(D,\omega \right)=\overset{}{\underset{{\mathbb{R}}^{3}}{\int }}dx\;dy\;dz{𝒦}_{{\text{PSF}}^{*}}\left(D,\omega \;|\;x,y,z\right)f\left(x,y,z\right).$$

**Figure 12:**
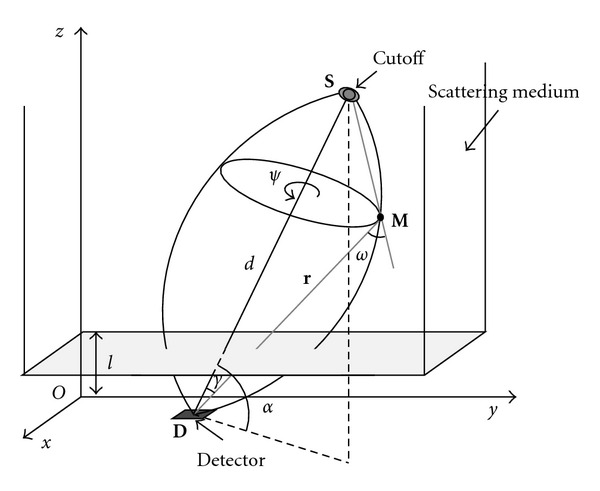
Torus surface of scattering sites for the case without collimator (reprinted with permission from [20]).

**Figure 13:**
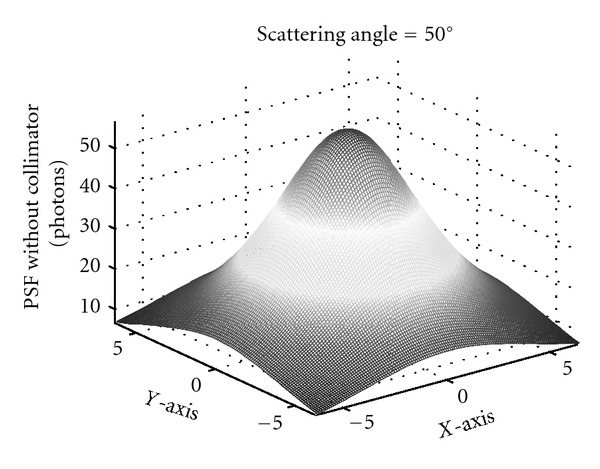
The PSF at a scattering angle of 50 degrees without collimator (reprinted with permission from [20]).

Unfortunately, the explicit form of *𝒦*_PSF*_(**D**, *ω* | *x*, *y*, *z*) is too complicated to yield a simple interpretation and will not be addressed here. However, this PSF, although no longer a computable function, is in fact an integral of the electronic density over the surface of a torus of revolution whose axis is the line connecting the point source to the detection point (Figure 12). The shape of the PSF is now completely different compared to the collimated geometry (Figure 13).

**Figure 14:**
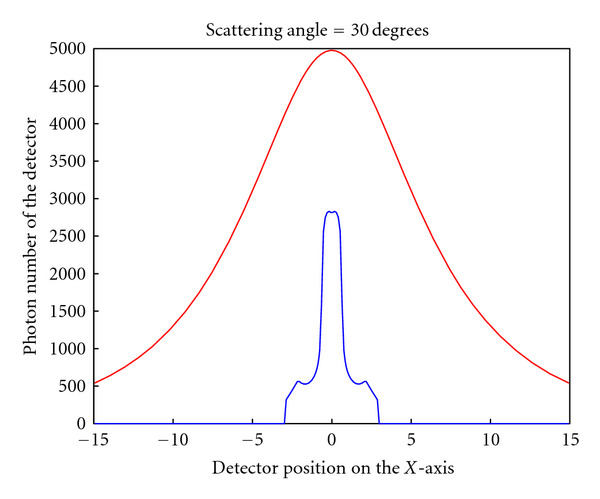
Comparison of the PSF with (lower blue line) and without collimator (upper red line), (reprinted with permission from [20]).

To compare with the collimated detector geometry, Figure 14 shows the computed PSFs for both cases at a scattering angle of 30 degrees. The PSF without collimator is about 12 times “stronger” than that with collimator (approximatively 3200 counts without collimator compared to approximatively 250 counts with collimator; see [38]).

**Figure 15:**
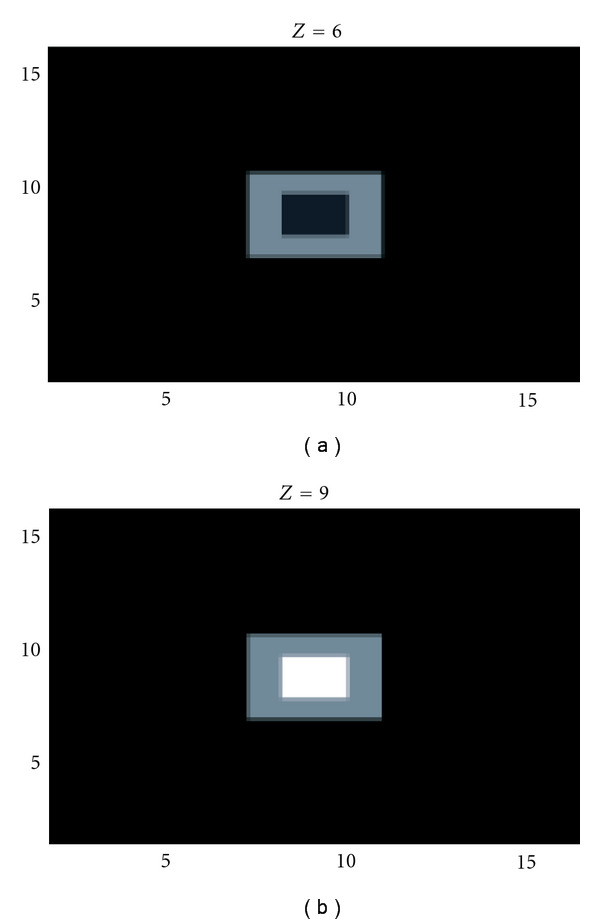
Two representative slices of the original object illustrating two transaxial slices corresponding to the 6th and 9th planes, respectively (reprinted with permission from [20]).

To demonstrate the viability of this idea, we have used data generated for a simple object and applied classical algebraic reconstruction methods. We have taken a simple source immersed in a cubic scattering medium. The source itself consists of two concentric cubes with different activity concentrations (Figure 15).

**Figure 16:**
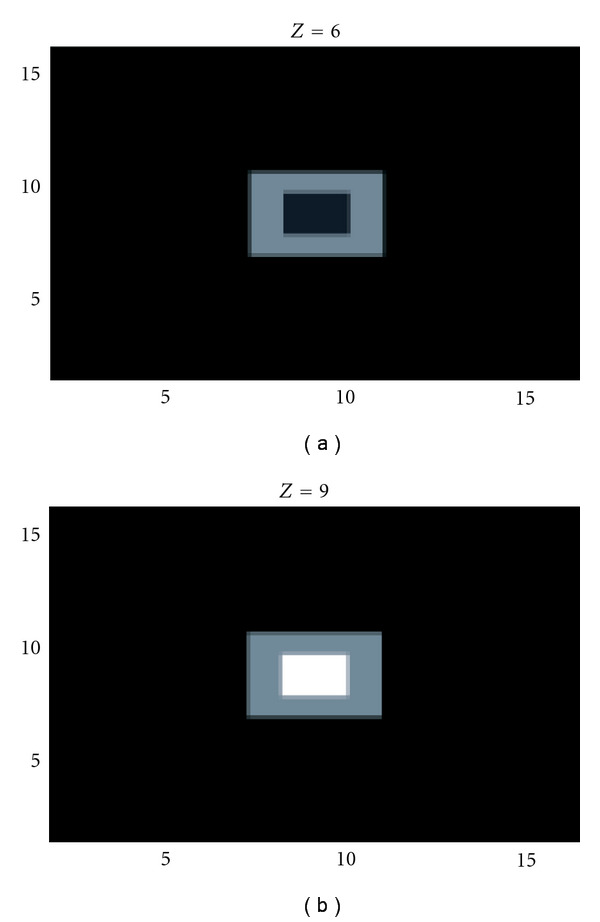
Two representative slices of the reconstructed images corresponding to the object shown in Figure 15 (reprinted with permission from [20]).

A 256 × 256 pixels detector is placed on the *xy* plane. The pixel size is 0.4 × 0.4 mm^2^. The scattering medium is a rectangular box of dimensions 30 cm by 30 cm by 15 cm, which is at a distance of 1 cm above the planar detector. The electronic density inside the scattering medium is *n*_*e*_ = 3.341023 electrons/cm^3^ since most biological tissues have an electronic structure close to that of water. The radionuclide used in this simulation is ^99*m *^Tc, which emits photons at an energy of 140.1 keV. The scattering medium is discretized with 13 voxels in *x* and *y* axis directions and with 9 voxels in *z*-axis direction. The detector is reduced to 13 × 13 pixels. We construct the weight matrix of the medium by calculating from our previous models, for each point of the mesh, the PSF of the detector at the different scattering angles. The reconstruction is carried out using the conjugated gradient method with positivity constraint; see Figure 16.

These results are an incentive to pursue the development of this imaging modality. 

## 4. Compton-Scattered Radiation Imaging in Two Dimensions

### 4.1. Image formation-Compounded V-Line Radon Transform (CVLRT)

**Figure 17:**
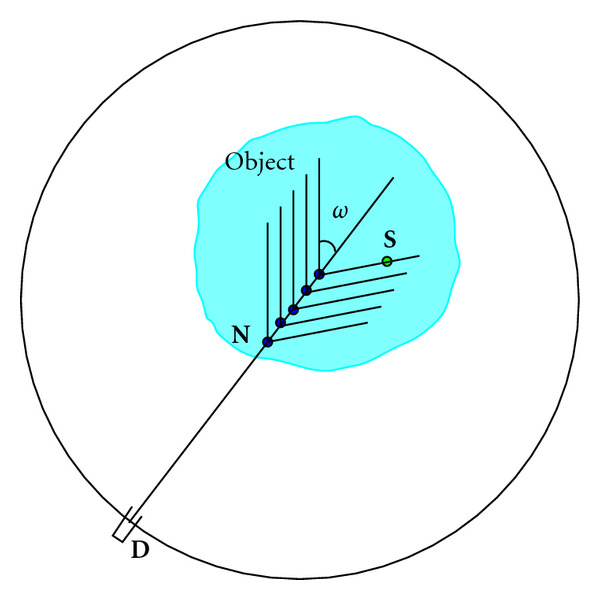
Compounded V-Line projection.

In this section, we discuss the transposition of the previous concept into a two-dimensional world. This passage entails a new form of the compounded conical projection, which is called now the compounded V-line projection, since the two-dimensional version of the cone surface is now just a geometric figure made up of two half-lines forming a letter V. This concept is illustrated in Figure 17.

 Inspection shows that each compounded V-Line projection depends on three parameters: two parameters for the line, and *ω* the scattering angle. Thus, an attempt to determine simultaneously the electron density *n*_*e*_(*x*, *y*) and the radioactivity density of the object with the totality of the compounded V-line projections in the plane will not be successful for lack of sufficient data. So we will examine only the case of constant *n*_*e*_(*x*, *y*) within the hypotheses adopted in the previous section.

**Figure 18:**
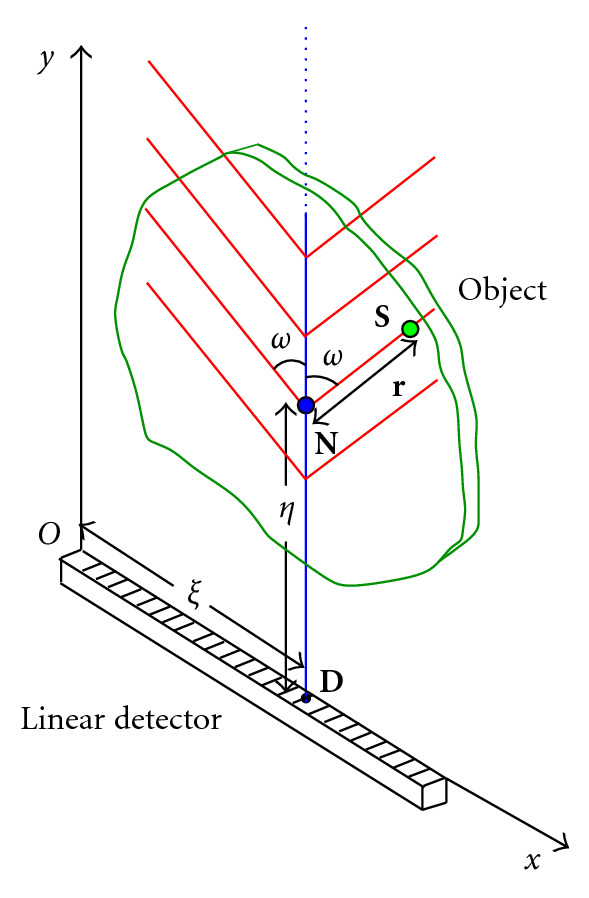
Setup and parameters of the compounded V-line Radon transform (CVLT).

This imaging process concerns two-dimensional structures in biomedical imaging, in which a radiotracer has been injected and maintained on a support.  Figure 18 shows the corresponding setup with a linear SPECT gamma camera for data acquisition.

In the image formation process, the compounded conical Radon transform is now replaced by the compounded V-line Radon transform. We give now the expression of the photon flux density at detecting site **D** for a scattering angle *ω* as in the previous section. Physical densities used here are actually derived from their three-dimensional values since we are dealing with real three-dimensional phenomena which are now restricted to a plane. They will appear with a star in formulas, for example, *n*_e_*(*x*, *y*) in lieu of *n*_*e*_(*x*, *y*, *z*). Let $\hat{f}(D,\tau )=\hat{f}(\zeta ,\tau )$ be the registered photon flux density, it is given by

(18) $$\;\;\begin{array}{cc} \hat{f}\left(\zeta ,\tau \right) & =K^{*}\left(\tau \right)\overset{\infty }{\underset{0}{\int }}\frac{d\eta }{\eta }\overset{\infty }{\underset{0}{\int }}\frac{dr}{r}\\ & \;\times [f\left(\zeta +r\sin\;\omega ,\eta +r\cos\;\;\omega \right)\\ & \;\;\;+f\left(\zeta -r\sin\;\omega ,\eta +r\cos\;\;\omega \right)], \end{array}$$

where *τ* = tan *ω*, K*(*ω*) = *πr*_*e*_^2^*n*_e_*𝒫(*ω*), the Compton kinematic factor restricted to two dimensions, 1/*η* and 1/*r* are the photometric propagation factor in two dimensions as opposed to the usual inverse square of the distance rule given in three dimensions. We call $\hat{f}(D,\omega )$ the compounded V-line Radon transform (CVLRT) of *f*(*x*, *y*). Inversion of this transform would allow the reconstruction of *f*(*x*, *y*) under the assumptions cited above. 

### 4.2. Inversion of the Compounded V-Line Radon Transform

The inversion procedure can be performed in two steps; see [41]. We present it with some details since it appears for the first time.

(i) First, let us define

(19) $$\overset{\infty }{\underset{0}{\int }}\frac{d\eta }{\eta }\;\;f\left(\xi_{\pm },\eta +r\cos\;\;\omega \right)=h\left(\xi_{\pm },r\cos\;\;\omega \right),$$

where *ξ*_±_ = (*ζ* ± *r*sin*ω*). Then, this problem is now shifted to the problem of a simple V-line Radon transform (VLRT) with the integration measure *dr*/*r* on the function *h*(*ξ*, *r*cos  *ω*). Hence, the inversion problem of the CVLRT is that of the inversion of VLRT for *h*, followed by the extraction of *f* from (19),

(20) $$\begin{array}{cc} & \hat{f}\left(\zeta ,\omega \right)\\ & =K^{*}\left(\omega \right)\overset{\infty }{\underset{0}{\int }}\frac{dr}{r}\\ & \;\times \left[h\left(\zeta +r\sin\;\omega ,\eta +r\cos\;\;\omega \right)+h\left(\zeta -r\sin\;\omega ,\eta +r\cos\;\;\omega \right)\right]. \end{array}$$

The inversion formula for the VLRT can be worked out following [42]. It yields

(21) $$\begin{array}{cc} & h\left(x,y\right)\\ & =\frac{y}{\pi }\overset{\pi }{\underset{0}{\int }}d\tau \\ & \;\times \left(\;\text{P}.\text{V}.\;\int_{\mathbb{R}}d\zeta \left(\frac{1}{\zeta -x-y\tau }+\frac{1}{\zeta -x+y\tau }\right)\frac{\partial \hat{f}\left(\zeta ,\tau \right)}{\partial \zeta }\right). \end{array}$$

  (ii) Now, knowing *h*(*x*, *y*), *f* is the solution of the convolution equation

(22) $$h\left(x,y\right)=\overset{\infty }{\underset{0}{\int }}\frac{d\eta }{\eta }f\left(x,\eta +y\right).$$

Thus, *f*(*x*, *y*) can be extracted by Fourier techniques [43]. Let

(23) $$\begin{array}{c} f\left(x,y\right)=\int_{\mathbb{R}}dke^{2i\pi ky}\;\bar{f}\left(x,k\right),\\ \overset{\infty }{\underset{0}{\int }}\frac{d\eta }{\eta }e^{-2i\pi k\eta }=-\left[\log\;2\pi \left|k\right|+\gamma +i\frac{\pi }{2}\mathrm{sgn}\;k\right] \end{array}$$

be the relevant Fourier transforms and *γ* = 0.57721566... the Euler's constant, then (22) becomes

(24) $$h\left(x,y\right)=\int_{\mathbb{R}}dke^{2i\pi ky}\;\bar{f}\left(x,k\right)\left(-\right)\left[\log\;2\pi \left|k\right|+\gamma -i\frac{\pi }{2}\mathrm{sgn}\;k\right].$$

The recovery of *f*(*x*, *y*) is achieved by inverse Fourier transform

(25) $$f\left(x,y\right)=\int_{\mathbb{R}}dqe^{2i\pi qy}\frac{\int_{\mathbb{R}}dze^{-2i\pi zq}h\left(x,z\right)}{\left(-\right)\left[\log\;2\pi \left|q\right|+\gamma -i\left(\pi /2\right)\mathrm{sgn}\;q\right]}\;,$$

as *h*(*x*, *z*) is known for all *z* ∈ ℝ.

Formula (21) lends itself to the derivation of a filtered back-projection (FBP) method for image reconstruction. This is because its structure is basically the one found in the standard Radon transform. Essentially, the procedure consists of the sum of two filtered back-projections on two half-lines forming the letter V. This is at first not obvious at all since in general the inverse of the sum of two operators is not necessarily the sum of their inverses. The advantage of the filtered back-projection inversion formula is that it can be implemented by fast algorithms. Simulation results are presented next. 

### 4.3. Numerical Simulations

**Figure 19:**
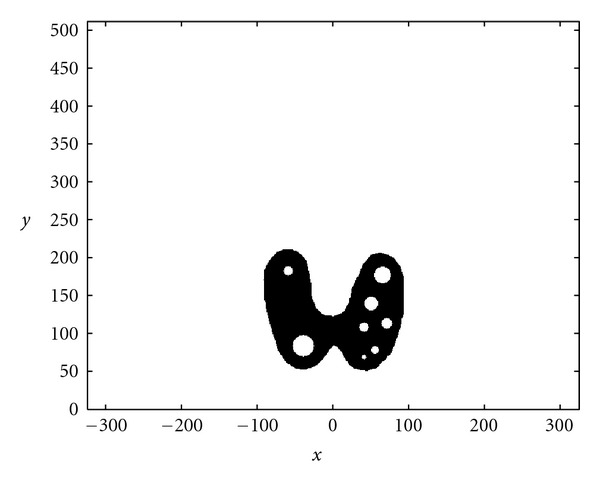
Original thyroid phantom.

**Figure 20:**
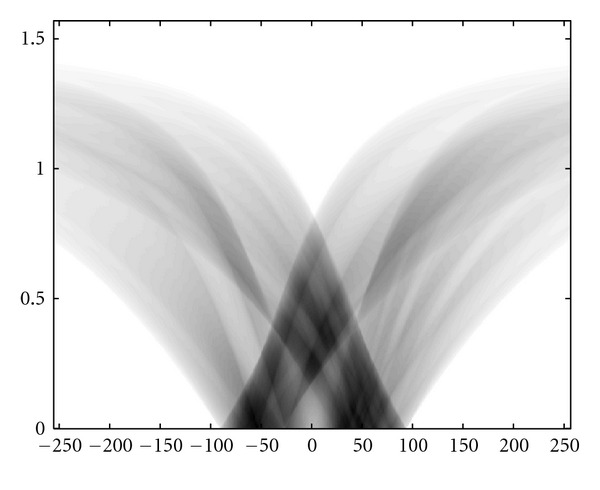
The *T𝕍* transform of the thyroid image shown in Figure 19 with *dω* = 0.0025 rad.

**Figure 21:**
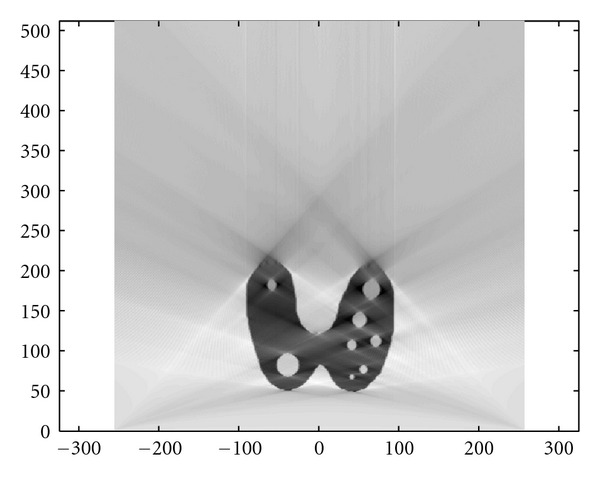
FBP-IM reconstruction of the thyroid phantom with *dω* = 0.0025 rad.

**Figure 22:**
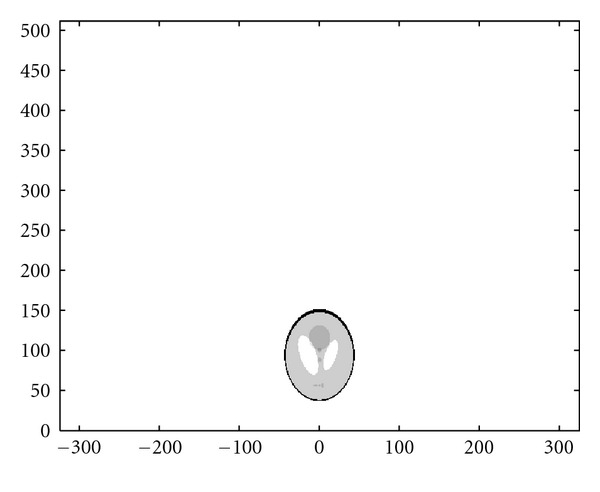
Shepp-Logan phantom.

**Figure 23:**
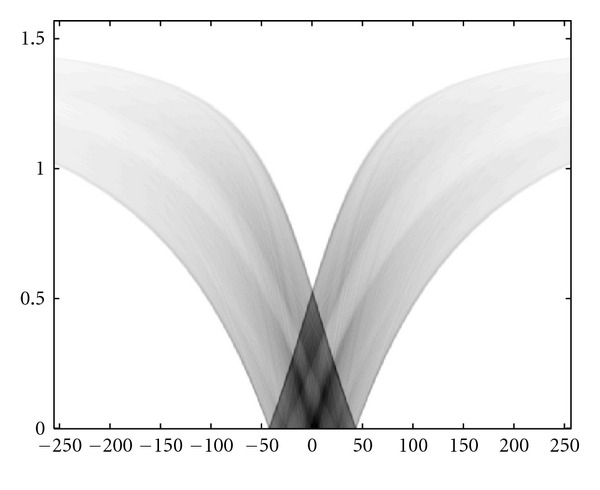
The *T𝕍* transform of the Shepp-Logan image shown in Figure 19 with *dω* = 0.0025 rad.

**Figure 24:**
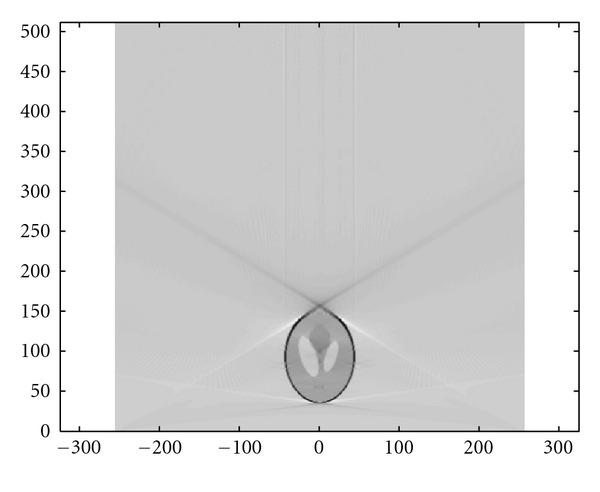
FBP-IM reconstruction of the Shepp-Logan phantom with *dω* = 0.0025 rad.

We present now the results of numerical simulations. The original image (Figure 19) of size 512 × 512 of length units is a thyroid phantom presenting with small nodules.  Figure 20 shows the CVLT transform of a thyroid phantom with angular sampling rate *dω* = 0.005 rad and 314 projections (*π*/2/0.005 = 314) which are the images of Compton-scattered radiation on the camera in terms of the distance *ξ* and the scattering angle *ω*. The reconstruction using FBP is given in Figure 21. The artifacts are due to the limited number of projections. Moreover, back-projection on V-lines generates more artifacts than back-projection on straight lines, because of more spurious line intersections. As our numerical results are based on the discretization of the inverse formula, a choice of a smaller discretization step *dω* would improve image quality. This is indeed a well-established fact and in agreement with the improved sampling resulting from the increase of data collected at more values of the scattering angle *ω*. Despite these limitations, the small structures in the object are clearly reconstructed. This result illustrates the feasibility of the new imaging modality, for which the main advantage resides in the use of a * one-dimensional nonmoving* Compton camera for * two-dimensional* imaging [42]. To show the possible medical application of this imaging modality, we have also presented the simulations on a Shepp-Logan phantom as a second example; see Figures 22, 23, and 24. 

## 5. Conclusion

The aim of this paper on Compton-scattered radiation emission imaging is to review the progress and development steps in this field during the last decade. The theoretical work is based on the inversion of generalized (and compounded) Radon transforms defined on cone surfaces in three dimensions and on V-lines in two dimensions. Numerical simulations appear to support the viability of the corresponding imaging process. The natural next research step to undertake is to study how this imaging principle behave under realistic operating conditions, namely

- how does it react to energy uncertainty measurements,
- how the CCRT inversion is modified if the electron recoil is not negligible,
- how to include the imperfections of the detection system (collimator response and electronic system response),
- how to account for limited statistics and random noise in typical real data acquisition situations.

In spite of these open questions, we still believe that this imaging technique has a strong potential as a future variant of the SPECT modality as soon as high performance detectors with very fine energy resolution are made available and accessible on a large scale. The future of scattered radiation emission imaging lies perhaps in its foremost advantage; it can provide a three-dimensional image reconstruction without having to move or displace the gamma camera (or planar detector). As discussed earlier, the usual rotation angle of an SPECT camera is now replaced with the Compton scattering angle. Thus, in scattered radiation emission imaging, the gamma camera is motionless and as such it does not require a heavy mechanical apparatus to accurately rotate the gamma camera around an object. It will be therefore less cumbersome and more convenient to use under stringent space conditions. In the end, it has led to a new concept of high energy resolution photon detector, a new development in data acquisition, and new image reconstruction methods derived from the compounded conical Radon transform and compounded V-line Radon transform. Applications in two- or three-dimensional imaging are possible particularly in medicine for clinical diagnosis and treatment planning. Finally, the tantalizing subject of identification via the concept of compounded conical projection looks very exciting and motivates future research in this direction.
